# Determinants of modern family planning use among women of reproductive age in the Nkwanta district of Ghana: a case–control study

**DOI:** 10.1186/1742-4755-11-65

**Published:** 2014-08-13

**Authors:** Sebastian Eliason, John K Awoonor-Williams, Cecilia Eliason, Jacob Novignon, Justice Nonvignon, Moses Aikins

**Affiliations:** 1 Department of Community Medicine, School of Medical Sciences, University of Cape Coast, Cape Coast, Ghana; 2 Regional Health Directorate, Ghana Health Service, Upper East Region, Bolgatanga, Ghana; 3 Department of Adult Health, School of Nursing, College of Health Sciences, University of Ghana, Legon, Ghana; 4 Department of Economics, University of Ibadan, Ibadan, Nigeria; 5 Department of Health Policy, Planning and Management, School of Public Health, College of Health Sciences, University of Ghana, Legon, Ghana

**Keywords:** Family planning, Contraception, Reproductive age, Case-control study, Ghana

## Abstract

**Background:**

Average contraceptive prevalence rate in the Nkwanta district of Ghana was estimated to be 6.2% relative to the national average at the time, of 19%. While several efforts had been made to improve family planning in the country, the district still had very low use of modern family planning methods. This study sought to determine the factors that influenced modern family planning use in general and specifically, the factors that determined the consistently low use of modern family planning methods in the district.

**Methods:**

A case–control study was conducted in the Nkwanta district of Ghana to determine socio-economic, socio-cultural and service delivery factors influencing family planning usage. One hundred and thirty cases and 260 controls made up of women aged 15–49 years were interviewed using structured questionnaires. A logistic regression was fitted.

**Results:**

Awareness and knowledge of modern family planning methods were high among cases and controls (over 90%). Lack of formal education among women, socio-cultural beliefs and spousal communication were found to influence modern family planning use. Furthermore, favourable opening hours of the facilities and distance to health facilities influenced the use of modern contraceptives.

**Conclusion:**

While modern family planning seemed to be common knowledge among these women, actual use of such contraceptives was limited. There is need to improve use of modern family planning methods in the district. In addition to providing health facilities and consolidating close-to-client service initiatives in the district, policies directed towards improving modern family planning method use need to consider the influence of formal education. Promoting basic education, especially among females, will be a crucial step as the district is faced with high levels of school dropout and illiteracy rates.

## Background

Uptake of modern family planning (FP) methods remains low in Sub-Saharan Africa (SSA) including Ghana. This may be associated with high incidence of unintended pregnancies, unsafe abortions, and maternal deaths [1,2]. The United Nation’s Millennium Development Goal (MDG) 5 aims at improving maternal health and reducing maternal mortality by three quarters between 1990 and 2015. An important intervention towards achieving this target is the promotion of modern family planning (FP) among women in Sub- Saharan Africa (SSA) [1,3]. Evidence exists that if couples can space their pregnancies by at least two years apart through the use of family planning, up to 35% of maternal deaths and up to 13% of child mortalities could be averted [4-6], whilst 25% of under – five mortalities could be averted if birth intervals were at least three years [5].

The cost-effectiveness of family planning in poverty reduction and socio-economic development in the developing world cannot be disputed [7,8]. Evidence from Bangladesh shows that long-term investment in an integrated family planning and maternal and child health (FPMCH) programme contributed to improved economic security for families, households, and communities through larger incomes, greater accumulation of wealth, and higher levels of education [8]. Further evidence showed that every dollar spent on family planning saved at least US$4 that could have been spent treating complications from unintended pregnancies and saved governments up to US$31 in health care, water, education, housing, sewers, and many more [9,10]. Thus, if individuals and couples, especially in the developing world, planned their pregnancies and families, with improved use of contraception, they are more likely to have fewer and healthier children which will reduce the socio-economic burden on them and allow more investment in each child’s care and education, helping to break the cycle of poverty.

Several studies have been conducted on determinants of family planning use. In some cases strong associations have been established between family planning use and some socio-demographic, socio-cultural and socio-economic characteristics of women and significant others. Depending on the context and setting in which the studies were conducted results were either consistent or contrasting. Studies have revealed that, use of family planning methods was found more in women of higher age group [11,12]. Education has also been observed in many other studies to be associated with contraceptive use [11,13-15]. Women with at least primary level of education are 8-10% more likely to be using a modern, or any method of contraception compared to those with no education. Secondary education increases the probability of using contraceptives in the range of 14-17%, and post-secondary education increases the probability to the range 16-20% [16]. High parity is associated with family planning use [11,12,15]. Family planning was more common among women with higher household income and highest wealth quintile [11,15].

In an analysis of 10,204 women from the 2011 demographic and health survey data of Ethiopia, it was reported that, being employed, being in a monogamous relationship, attending community conversation and being visited by a health worker at home increased the likelihood of using modern contraception [15]. They also observed on the contrary that, living in rural areas, older age, being in polygamous relationship, being Muslim, and witnessing one’s own child’s death were associated with lower odds of adopting modern contraceptive [15].

Stephenson and Hennink [17] noted that family planning use and service utilization was associated with socio-economic, demographic, cultural and health experience factors at the individual and household levels. They noted that increasing parity and approval by partner and significant others like mother –in-law were associated with increased family planning service utilization. They also observed that the further away family planning facilities were from women, the lower the tendency to access services [17]. Those who communicated with their spouses had elevated odds of using family planning. The assumption was that spousal communication was associated with subsequent family planning exposure, which in turn led to family planning use [18].

Family planning is integrated into the reproductive and child health services at all levels of the health delivery system in Ghana. Health service delivery in Ghana follows a three-tier arrangement: primary, secondary and tertiary levels. The primary level incorporates a Community Based Health Planning and Service (CHPS) system that ensures health provision at the door step of the people. Family planning services are free, but the commodities are not, though they are very highly subsidized. Strong advocacy is however ongoing, to make commodities free in the country. Doctors, midwives and community health nurses are responsible for family planning service provision in the country.

Ghana’s fertility rate was estimated to be at 4 per woman in 2008 which was a reduction from 5.2 in 1993 and 4.4 in 2003 [19]. About 17% of married women use modern contraceptive methods in Ghana while about 24% use any contraceptives, including traditional contraceptives [19]. This suggests that the use of contraceptive methods, especially the modern ones, still remain low among women in Ghana. It is estimated that, Ghana’s family planning unmet need increased from 30% in 2003 to 34% in 2008 [19]. It is therefore imperative to establish and understand the factors that serve as barriers to the use of contraceptives and available family planning services.

The Nkwanta district is located in the Volta region of Ghana and is considered to be one of the poorest and most remote districts in the region. It has a population of 117,878. The district boasts of 13 health facilities including 2 hospitals, health centres, maternity homes and community based health planning and services (CHPS) compounds. The health workforce in the district is inadequate and weak. The Doctor: Patient ratio is 2: 117,878 and the Nurse: Patient ratio also is 1:7,000.

Family planning is integrated into the reproductive and child health programme of the district. Routine service-provision data, as well as survey data from 2000 had consistently indicated low prevalence of modern contraceptive use [20] compared to all the other districts in the Volta region. According to the annual reports of the District Health Directorate, the average contraceptive prevalence rates computed for the district increased from 3.7% in 1999 to 6.2% in 2004 [21] relative to the overall national average at the time of 19% and national rural average of 14% [22]. This trend had become very worrying to the district. The aim of this study was to determine the factors that influenced modern family planning use in general and specifically, the factors that determined the consistently low use of modern family planning methods in the district. This would inform more committed efforts at improving family health and wealth in the region. Field work was undertaken between July and August 2004.

## Methods

### Study methodology

A case control study method was employed because family planning use had been consistently low over a long period compared to the other districts in the region and in the country; funding for study was limited and results of the study were needed quickly by the district to put in necessary interventions. A 2:1 matching was employed in this study, where two control persons were sought for every case person.

### Study and target populations

Cases: The study population included all registered modern family planning users aged 15–49 years in the Nkwanta district. The target population included all women aged 15–49 years living in Nkwanta district and currently using any modern contraceptive method, and all women in the specified age group who were not practicing any modern method of contraception, but whose partners were practicing modern male contraception. All women aged 15–49 years who were practicing traditional methods of contraception were excluded because the problem under study was low use of modern family planning.

Controls: Study population included all women aged 15–49 years living in Nkwanta district who had never practiced any modern family planning method. The target population included women aged 15–49 years (matched for age group, marital status, place of residence) living in Nkwanta district who had never used any modern contraceptive method. Women aged 15–49 years who had practiced modern family planning in the past, but who were currently not practicing any modern contraception, were excluded. Women who were currently practicing traditional methods of contraception were also excluded.

### Sample size

The sample size, *n* necessary to estimate the odds ratio (OR) to within Ɛ of OR, with probability 1- α, is given by [23]:

n=z1−α221/p1*1−p1*+1/1−p2*)/ln1−ϵ2

Where P_1_^*^ and P_2_^*^ are the probabilities of exposure given that a person practices family planning or not, respectively [23], with

$$p_{1}*=\left[\left(\mathrm{OR}\right)p_{2}*\right]/\left[\left(\mathrm{OR}\right)p_{2}*+\left(1-p_{2}*\right)\right]$$

To estimate the population odds ratio (OR) to within Ɛ =25% of its true value, 2, given that the exposure rate computed from a pilot survey was 0.30, the sample size, *n* was = 408; But in this study, two controls were matched for each case i.e. *n*_control_/*n*_case_ = 2. The exact number of cases and controls were derived as follows: *n*_*cases*_ + *n*_*controls*_ = 408 (*n*_*controls*_ = 2*n*_*cases*_). The number of cases *n*_*cases*_ was 136; whilst the number of controls *n*_*control*_ was 272.

### Identification of cases and controls

Since there was a district register of all women practicing modern family planning, selection of cases, was initially by a stratified sampling procedure as follows:

Stratification of all women aged 15–49 years currently practicing modern family planning, and have their names in the district’s family planning register, into the following age groups- (15-19 yrs), (20-29 yrs), (30-39 yrs) and (40-49 yrs).

Using proportional allocations sample sizes were assigned to each age stratum.

Simple random sampling was used to select the pre-determined number of subjects for each age stratum from the stratified register. Subjects were to be traced to their household using the addresses given in the district family planning register.

At this point, it was realised that most of the addresses provided in the registers were unreliable because of poor household address systems, some evidence of covert use of family planning and culturally sensitive nature of family planning in the district.

The cases were therefore selected through community health officers (CHOs) who were requested to recruit as many women as possible practicing family planning from the various sub-district lists extracted from the district register. This was much easier and suitable because the CHOs knew the clients personally and also knew the households in which they lived. Four (4) out of the five (5) sub-districts were selected (i.e. Kpassa, Brewaniase, Tutukpene, and Nkwanta) because they had CHOs at the health centres or CHPS centres who would help recruit cases. The clients were invited to report at the various health facilities to be screened. Once they fell within the inclusion criteria, they were recruited into the study. Despite the challenges and limitations faced in sampling, a total of 130 cases - Kpassa (30), Brewaniase (23), Tutukpene (32), and Nkwanta (45) - were recruited, giving a response rate of 95.6%.

Control persons were recruited from the same communities where case persons resided, by matching for age, marital status and place of residence. Active search for controls was done starting from the households of the cases. If suitable controls were found for the cases in the households, they were recruited. If controls were not found within the households, they were sought for in the adjacent households till suitable controls were found (two controls per case). A total of 260 controls were recruited.

### Data collection

To formalize the data collection process and to ensure reliable responses, consents were sought from the District Chief executive, Chiefs and opinion leaders within the second week. The objectives and likely outcomes were explained to these individuals and culturally sensitive issues in the survey instrument discussed and restructured in a way that would minimize the respondents’ anxiety and at the same time provide the most reliable responses.

Similarly, qualified research assistants were recruited for the purposes of effective data collection. Within the third week, eight (8) research assistants who were fluent in both English language and the local dialects were recruited from the district and trained to do survey data collection using the standardized questionnaire. During the training, the concept of case–control studies, the objectives and methodology of the study were also discussed in detail and explained using the training manual. Each of the Research Assistants was made to translate the questionnaire into local language in the presence of all the others to ensure that he or she understood the questions. Where the translation was not well done, he or she was helped by experts to do them right.

All the questionnaires together with the methodology were pre-tested by the Research Assistants (RAs) at Korantang, a community outside the study area but with similar characteristics like Nkwanta. The necessary corrections and reviews were made before they were finally taken to the field. These activities were carried out in the fourth week.

Interview survey technique using the same structured questionnaires was employed to obtain quantitative data from both cases and controls. Information about the following exposure or explanatory variables was obtained from both cases and controls. The exposure variables were broadly classified into three categories, namely: socio-demographic, socio-economic, socio-cultural and service factors. The socio-demographic characteristics included parity and ethnicity whilst the socio-economic variables included the educational levels and employment status of respondents and their partners, household income, living conditions, and type of employment. The socio-cultural variables included religion, religious approval of FP, discussion with parents and partner about FP, preference for a given number of children, beliefs and myths about side effects of FP, living with significant others and polygamous partner. The service factors included method availability, cost of methods, facility waiting times, facility opening hours, distance of facility from community and time constraints and attitudes of health workers.

The Research Assistants were divided into two (2) groups of four (4). One group was assigned the cases and the other group, the controls. About fifteen (15) to twenty (20) minutes was spent per subject. The interviews were conducted confidentially by the trained assistants at the health facilities and in the communities. Three (3) weeks was assigned for the data collection.

### Quality control

Before each interview, the research assistant introduced him/herself to the respondent, and the objectives and the possible outcomes explained to them. It was also explained that the answers that they provided were going to be kept confidential. After each subject was interviewed, the questionnaires were scrutinized for completeness and consistency. Where incompleteness, errors and inconsistencies were observed, the necessary corrections were ensured before the women were allowed to leave.

### Ethical considerations

The study was subjected to ethical clearance from the Noguchi Memorial Institute for Medical Research at the University of Ghana. Informed consent was also sought from the subjects involved in the data collection. Further, confidentiality of collected information was ensured by clients not providing names or any identities. Subjects had the right to opt out of the study any time they desired. Subjects were made comfortable and protected from any physical and mental harm in the course of the data collection. Initial findings of the study were presented to the district.

### Data analysis

All completed interviews were rechecked for consistency and completeness. Coding and computerisation were done after creation of data analysis fields in Epi Info statistical software package version V3.0. The information was transferred to STATA statistical software version 7.0 for statistical analysis at the time. The data was re-analysed using the STATA version 11.2. Simple frequencies were used to analyse respondents’ background data, whilst logistic regression models (bivariate and multivariate) were employed to test level of significance and association between the dependent and exposure or explanatory variables. The dependent variable used in the logistic regression analyses was “current use of modern family planning methods among respondents”. Modern family planning includes reversible (pills, injectables, condoms, intrauterine device, implants, lactational amenorrhoea) and irreversible (vasectomy and tubal ligation) contraceptive methods. The variable was measured as a dummy binary variable, with 1 representing usage and 0 representing non-usage.

## Results

### Background characteristics of study women

Most of the cases (63/130) and controls (126/260) were in the 20–29 year group. Most Tawere also from Nkwanta sub-district and were married. Amongst the cases, majority (39.2%) had attained juniour secondary/middle school education, whilst majority of the controls (50.8%) had had no formal education. Majority of the cases (23.8%) belonged to the Ewe ethnic group whilst Konkombas, dominated the control group (23.1%). Christians and farmers dominated the samples of cases and controls Table 1.

**Table 1:** Characteristics of study participants

**Characteristic**	**Cases**	**Controls**	**Total**
**Sub-district:**	**Frequency (%)**	**Frequency (%)**	**Number**
Brewaniase	23	46	69
Kpassa	30	60	90
Nkwanta	45	90	135
Tutukpene	32	64	96
Total	130	260	390
Age:			
15-19	2	4	6
20-29	63	126	189
30-39	52	104	156
40-49	13	26	39
Total	130	260	390
Marital Status:			
Married	127	254	381
Not married	3	6	9
Total	130	260	390
Educational level:			
None	42 (32.3)	132 (50.8)	174
Primary	30 (23.1)	49 (18.8)	79
JSS/MLSC	51 (39.2)	53 (20.4)	104
SSS/Vocational	6 (4.6)	22 (8.5)	28
Tertiary	1 (0.8)	4 (1.5)	5
Total	130 (100)	260 (100)	390
Ethnicity:			
Ewe	31 (23.8)	50 (19.2)	81
Konkomba	24 (18.5)	60 (23.1)	84
Adele	15 (11.5)	40 (15.4)	55
Ntrobo	23 (17.7)	17 (6.5)	40
Achode	10 (7.7)	42 (16.2)	52
Basare	10 (7.7)	21 (8.1)	31
Other	17 (13.1)	30 (11.5)	47
Total	130 (100)	260 (100)	390
Employment status:			
Farming/animal husbandry	47 (36.,2)	121 (46.5)	168
Trading	33 (25.4)	78 (30.0)	111
Civil servant	5 (3.8)	11 (4.2)	16
Artisan	38 (29.2)	41 (15.8)	79
Unemployed	4 (3.1)	7 (2.7)	11
Others	3 (2.3)	2 (0.8)	5
Total	130 (100)	260 (100)	390
Religion:			
Islam	13 (10.0)	20 (7.7)	33
Christian	95 (73.1)	215 (82.7)	310
Traditional	15 (11.5)	13 (5.0)	28
No religion	7 (5.4)	12 (4.6)	19
Total	130 (100)	260 (100)	390

### Knowledge and awareness of family planning

A little over 90% of both cases (93.8%) and controls (91.5%) knew at least a method of modern contraceptive. Injectable was the most known modern method of family planning amongst both cases (93.1%) and controls (82.6%), followed by the pill (cases-86.9%; controls-65.9%). The diaphragm was the least known method amongst the cases (3.1%), whilst vasectomy or male sterilization was the least known amongst the controls (0.4%) (Table 2). Health workers were the main source of family planning information in the district for both cases and controls, followed by friends and relatives and then by radio. Newspaper sources contributed the least to family planning information in the district (Table 3).

**Table 2:** Knowledge of modern family planning methods

	**Cases**		**Controls**	
	**(Yes)**	**(No)**	**(Yes)**	**(No)**
**Method**	**Frequency (%)**	**Frequency**	**Frequency (%)**	**Frequency**
Female sterilization	30 (23.1)	100	5 (1.9)	253
Male sterilization	7 (5.4)	123	1 (0.4)	257
Pill	113 (86.9)	17	170 (65.9)	88
IUD	61 (46.9)	69	30 (11.6)	228
Injectables	121 (93.1)	9	213 (82.6)	45
Implant	101 (77.7)	29	51 (19.8)	207
Male condom	67 (51.5)	63	41 (15.9)	217
Female condom	67 (51.5)	63	4 (1.6)	254
Diaphram	4 (3.1)	126	3 (1.2)	255
None	8 (6.2)	122	22 (8.5)	236
	N = 130		N = 258^	

^missing values.

**Table 3:** Source of information on modern family planning methods

	**Cases**		**Controls**	
	**(Yes)**	**(No)**	**(Yes)**	**(No)**
**Source**	**Frequency (%)**	**Frequency**	**Frequency (%)**	**Frequency**
Radio	33 (25.4)	97	34 (13.2)	224
Television	10 (7.7)	120	8 (3.1)	250
Newspapers/posters/brochures	10 (7.7)	120	13 (5.0)	245
Health workers	101 (77.7)	29	218 (84.5)	40
Commercial/social club	10 (7.7)	120	5 (1.9)	253
Friends/relatives	83 (63.8)	47	36 (14%)	222
	N = 130		N = 258^	

^missing values.

### Determinants of family planning method use

At the time of the survey, Konkomba women showed significantly less odds of practicing modern family planning compared to women of other ethnic groups (OR = 0.51, 95% C.I.0.29 -0.90) in bivariate analyses. Women with no formal education were significantly less likely to practice modern family planning relative to those with some formal education (OR = 0.57, 95% C.I 0.37- 0.87). Similarly, women whose partners had no formal education were also significantly less likely to practice modern family planning (OR = 0.48, 095% C.I.0.29- 0.80). In multivariate analyses, Konkomba women still showed significantly less odds of practicing modern family planning (AOR = 0.25, 95% CI 0.07 -0.91) (Table 4).

**Table 4:** Logistic regression of modern family planning use on socio-demographic and socio-economic factors

	**Family planning**	**Bivariate**	**Multivariate**
		**O.R (95% C.I)**	**AOR (95% C.I)**
Parity<=4	Yes	1.00 (0.65, 1.65)	1.38 (0.41, 4.65)
	No	1.00	
Ethnic Konkomba	Yes	0.51 (0.29, 0.90)*	0.25 (0.07, 0.91)*
	No	1.00	
Educational level-respondent(none)	Yes	0.57 (0.37, 0.87)**	0.59 (0.18, 1.92)
	No	1.00	
Educational level-partner(none)	Yes	0.48 (0.29, 0.80)**	2.33 (0.62, 8.73)
	No	1.00	
Respondent employment- (none)	Yes	0.98 (0.64, 1.51)	0.70 (0.21, 2.28)
	No	1.00	
Partner employment-none	Yes	0.83 (0.55, 1.27)	1.80 (0.57, 5.70)
	No	1.00	

Notes: *Denote P-value < 0.05 and **P-value < 0.01.

As depicted in Table 5, women who discussed modern family planning with their partners were 4.67 times more likely to use modern family planning methods compared to those who did not discuss with their partners (OR = 4.67, 95% C.I 2.88-7.58) in bivariate analyses. Furthermore, women whose partners approved of modern family planning were 4.33 times more likely to use modern family planning than those whose partners did not approve (OR = 4.33, 95% C.I 2.55-7.36). In multivariate analyses, women who believed that modern family planning use made women promiscuous showed lower odds of practicing modern family planning compared to those without that belief (AOR = 0.06, 95% C.I 0.01- 0.47) Table 5.

**Table 5:** Logistic regression of modern family planning use on Socio-cultural factors

	**Family planning**	**Bivariate**	**Multivariate**
		**OR. (95% C.I)**	**AOR. (95% C.I)**
Religion-Islam	Yes	0.51 (0.22, 1.21)	1.15 (0.22, 5.91)
	No	1.00	
Woman can go out alone to hosp.	Yes	2.1 (0.84, 5.28)	1.23 (0.20, 7.44)
	No	1.00	
Approval of mother/father-in law/parent	Yes	0.96 (0.46, 1.99)	0.57 (0.21, 1.60)
	No	1.00	
Discussion with partner	Yes	4.67 (2.88,7.58)**	2.45 (0.79, 7.53)
	No	1.00	
Partner approval	Yes	4.33 (2.55,7.36)**	1.70 (0.48, 6.01)
	No	1.00	
Preference for less than 4 children by women	Yes	1.19 (0.72, 1.97)	3.54 (0.72, 17.34)
	No	1.00	
Preference for less than 4 children by partner	Yes	1 (0.56, 1.77)	0.33 (0.06, 1.84)
	No	1.00	
Women’s belief that use cause anger from God	Yes	0.85 (0.53, 1.38)	0.94 (0.25, 3.61)
	No	1.00	
Women’s belief that use makes woman unhealthy	Yes	0.74 (0.45, 1.21)	1.06 (0.23, 4.87)
	No	1.00	
Women’s belief that use makes woman promiscuous	Yes	0.72 (0.42, 1.22)	0.06 (0.01, 0.47)**
	No	1.00	
Partner’s belief that use causes anger from God	Yes	0.74 (0.44, 1.23)	1.83 (0.35, 9.51)
	No	1.00	
Partner’s belief that use makes woman unhealthy	Yes	0.88 (0.53, 1.45)	1.96 (0.38, 10.27)
	No	1.00	
Partner’s belief that use makes woman promiscuous	Yes	0.94 (0.57, 1.55)	2.48 (0.44, 13.84)
	No	1.00	

**Denote P-value < 0.01.

A family planning facility less than 5 km away was more likely to influence modern family planning use compared to if it was located more than 5 km away (OR = 3.63, 95% C.I 1.38- 9.55) (Table 6). Also, method availability showed greater odds of influencing modern family planning use compared to method non-availability (OR = 4.98, 95% C.I 2.55- 9.75). Favourable opening hours (five hours or more) of family planning facility significantly increased odds of modern family planning use compared to non-favourable opening hours (OR = 9.25, 95% C.I 4.50 -19.03)). Non- restriction of method use was found to be 1.75 times more likely to influence modern family planning use relative to situations with restrictions to method use (OR = 1.75, 95% C.I 1.03-2.96). In multivariate analyses however, favourable opening hours of family planning facility showed increased odds of influencing modern family planning use compared to non-favourable opening hours (AOR = 5.38, 95% C.I 1.36-21.29) (Table 6).

**Table 6:** Logistic regression of modern family planning use on Service factors

	**Family planning**	**Bivariate**	**Multivariate**
		**O.R (95% C.I)**	**AOR (95% C.I)**
Facility distance <5 km	Yes	3.63 (1.38, 9.55)**	1.65 (0.21, 13.11)
	No	1.00	
Methods readily available	Yes	4.98 (2.55, 9.75)**	1.67 (0.38, 7.38)
	No	1.00	
Methods expensive	Yes	1.68 (0.98, 2.87)	0.68 (0.17, 2.76)
	No	1.00	
Favourable opening hours	Yes	9.25 (4.50, 19.03)**	5.38 (1.36, 21.29)*
	No	1.00	
Waiting long hours	Yes	1.19 (0.72, 1.95)	0.40 (0.11, 1.40)
	No	1.00	
No restrictions	Yes	1.75 (1.03, 2.96)*	0.70 (0.21, 2.37)
	No	1.00	

*Denote P-value < 0.05 and **P-value < 0.01.

## Discussion

The findings of the study seem to suggest that knowledge of modern family planning methods is very high amongst both cases and controls as a little over 90% of them had knowledge of at least one modern contraceptive method. This result confirms what is reported in the Ghana Demographic and Health Survey (GDHS) [19] that about 98% of all women aged 15–49 years know at least one modern method of contraception.

Injectables were the most known method of modern contraception amongst both cases (93.1%) and controls (82.6%), followed by the pills and implants. This is contrary to the GDHS [19] where male condom was the most known method of modern contraception, followed by the pill and injectables. The injectables were well known because it is believed that it was suitable for covert users of modern contraceptives, especially in a community in which women had the belief that practice of modern family planning was associated with promiscuity (finding in this study). It is rather surprising that male condoms were found to be unpopular amongst women in the Nkwanta district. The patriarchal nature of the district [20] may be promoting the belief that family planning is a ‘woman’s business’ and therefore patronage of the use of male methods may not be prevalent.

The study suggested that the main source of family planning information was health workers in the district. This may be explained by the fact that health workers are accessible to the various communities in the district throughout the day because of the presence of the community based health planning and services (CHPS) system which is widespread in the communities and provide health service at the doorstep of the people. Also, the Nkwanta district generally has very poor radio and television reception hence making it difficult to obtain regular family planning information from such media.

As expected, the study found that partners’ consent to the use of modern family planning methods was very crucial to the success of any family planning intervention. The results showed that women who discussed family planning methods with their partners were more likely to use modern methods of contraception. This finding is consistent with empirical findings from other researchers in other countries [18,24-26]. For instance, Mekonnen and Worku [27] found that women who discussed family planning with their partners were 2.2 times more likely to use family planning.

Moreover, evidence from Nigeria revealed that husband opposition was the major reason for non-use of modern contraceptives [28]. It is speculated that some partners who do not approve of family planning use feared that their wives use of contraceptives would encourage promiscuity or would undermine their authority as heads of the household [29]. This belief was confirmed in this study and is a major barrier to modern family planning use in the district. It was revealed that women who had the belief that use of modern family planning promoted promiscuity had significantly lower odds of practicing modern family planning compared to women who did not have that belief (AOR = 0.06, 95% C.I 0.01- 0.47). It may be possible that the partners of these women may have influenced such belief.

The study also found that distance to family planning facilities influenced use of contraceptive methods. Specifically, communities with family planning facilities within a distance of less than 5 km away are more likely to use contraception as opposed to communities with facilities with a distance of more than 5 km. This finding contradicts that of a study in Nigeria in which 40% of women interviewed did not attend the clinic nearest to their homes and 90% of these women said they did so to get better service [30]. Favourable opening hours of the family planning facilities was also found to be strongly associated with modern family planning use. In the Nkwanta district, the Community Based Health Planning and Services (CHPS) initiative that seeks to bring health services to the doorstep of the people has improved accessibility to family planning services greatly. This was shown by the gradual increase in family planning coverage from 3.7% in 1999 to 6.2% in 2004. The finding in this study reinforces the need to promote and maintain the CHPS initiative in the district.

Educational attainments of both women and their partners were found to be very significant factors in the use of family planning methods. Unlike women with no formal education, women with some formal education were more likely to use modern methods of contraception. A similar relationship was established for their partners. Other researchers also found similar results in their studies [31-34]. Beekle and McCabe [31] found lack of formal education to strongly reduce modern family planning use. This does not come as much of a surprise as higher education attainment increases female decision making power and awareness of the benefits of good family planning practices [17]. This study found that, Konkomba women compared to women from the other ethnic groups in the district had lower odds of practicing modern family planning. Lower educational attainment [20] may explain this finding.

The results show that while family planning knowledge and awareness was widespread in the district, factors such as education, access to facilities and services and partner’s consent were significant determinants of modern family planning method use. Major determinants of low family planning use in the district, are low educational attainment of women in the district especially, the Konkomba women and strong socio-cultural beliefs amongst women, especially the belief that modern family planning use make women promiscuous. In addition to providing health facilities and consolidating the CHPS initiative in the district, policies directed towards improving modern family planning method use need to consider raising the levels of formal education. Promoting basic education, especially among females, will be a crucial step as the district is faced with high levels of school dropout and illiteracy.

The study was limited in the sense that while stratified sampling method was more preferred, the poor nature of household addresses in the district did not make this feasible to its full extent. Furthermore, the extent to which the findings from the study can be generalized is limited due to the various sample size adjustments performed. However, these limitations do not affect the reliability of the findings of the study.

## Conclusion

This case–control study sought to determine factors influencing the use of modern family planning in the Nkwanta district of Ghana, especially those that influence low use. The results show that, while family planning knowledge and awareness are wide spread in the district, factors such as low educational attainment of women in the district and widespread socio-cultural beliefs about modern family planning are major barriers to family planning use in the district.

In addition to providing health facilities and consolidating the CHPS initiative in the district, policies directed towards improving modern family planning method use need to consider raising the levels of formal education. Promoting basic education, especially among females, will be a crucial step as the district is faced with high levels school dropout and illiteracy.

## Competing interests

The authors declare they have no competing interests.

## Authors’ contributions

SE conceived the study, carried out data collection and participated in analysis and drafting the manuscript. JKAW participated in conceiving the study and reviewing the manuscript. CE participated in conceiving the study, developing data collection tools and reviewing the manuscript. JN(1) participated in analysis, drafting and reviewing the manuscript. JN(2) participated in analysis, drafting and reviewing the manuscript. MA provided general guidance and participated in drafting and reviewing the manuscript. All authors read and approved the final manuscript.
